# Sex-Specific Associations between Prenatal Exposure to Di(2-ethylhexyl) Phthalate, Epigenetic Age Acceleration, and Susceptibility to Early Childhood Upper Respiratory Infections

**DOI:** 10.3390/epigenomes8010003

**Published:** 2024-01-26

**Authors:** Sarah M. Merrill, Nicole Letourneau, Gerald F. Giesbrecht, Karlie Edwards, Julia L. MacIsaac, Jonathan W. Martin, Amy M. MacDonald, David W. Kinniburgh, Michael S. Kobor, Deborah Dewey, Gillian England-Mason, The APrON Study Team

**Affiliations:** 1 Department of Psychiatry and Human Behavior, The Warren Alpert Medical School at Brown University, Providence, RI 02903, USA; sarah_merrill@brown.edu; 2 Department of Medical Genetics, British Columbia Children’s Hospital Research Institute, University of British Columbia, Vancouver, BC V6T 1Z4, Canada; karlie.edwards@bcchr.ca (K.E.); julie.macisaac@bcchr.ca (J.L.M.); msk@bcchr.ca (M.S.K.); 3 Centre for Molecular Medicine and Therapeutics, Vancouver, BC V6H 0B3, Canada; 4 Faculty of Nursing, University of Calgary, Calgary, AB T2N 1N4, Canada; nicole.letourneau@ucalgary.ca; 5 Department of Psychiatry, Cumming School of Medicine, University of Calgary, Calgary, AB T2N 1N4, Canada; 6 Department of Pediatrics, Cumming School of Medicine, University of Calgary, Calgary, AB T2N 1N4, Canada; ggiesbre@ucalgary.ca (G.F.G.); dmdewey@ucalgary.ca (D.D.); 7 Owerko Centre, Alberta Children’s Hospital Research Institute, University of Calgary, Calgary, AB T2N 1N4, Canada; 8 Hotchkiss Brain Institute, Calgary, AB T2N 4N1, Canada; 9 Department of Psychology, Faculty of Arts, University of Calgary, Calgary, AB T2N 1N4, Canada; 10 Department of Community Health Sciences, Cumming School of Medicine, University of Calgary, Calgary, AB T2N 1N4, Canada; 11 Science for Life Laboratory, Department of Environmental Science, Stockholm University, 106 91 Stockholm, Sweden; jon.martin@aces.su.se; 12 Alberta Centre for Toxicology, University of Calgary, Calgary, AB T2N 1N4, Canada; amacdona@ucalgary.ca (A.M.M.); dkinnibu@ucalgary.ca (D.W.K.); 13 Department of Laboratory Medicine and Pathology, University of Alberta, Edmonton, AB T6G 2R3, Canada; 14 Program in Child and Brain Development, Canadian Institute for Advanced Research, Toronto, ON M5G 1M1, Canada; 15 University of Calgary, Calgary, AB T2N 1N4, Canada; apron@ucalgary.ca; 16 University of Alberta, Edmonton, AB T6G 2R3, Canada

**Keywords:** phthalates, DNA methylation, epigenetic clock, common cold, immunotoxicity

## Abstract

Di(2-ethylhexyl) phthalate (DEHP) is a common plasticizer that can affect immune system development and susceptibility to infection. Aging processes (measured as epigenetic age acceleration (EAA)) may mediate the immune-related effects of prenatal exposure to DEHP. This study’s objective was to examine associations between prenatal DEHP exposure, EAA at three months of age, and the number of upper respiratory infections (URIs) from 12 to 18 months of age using a sample of 69 maternal–child pairs from a Canadian pregnancy cohort. Blood DNA methylation data were generated using the Infinium HumanMethylation450 BeadChip; EAA was estimated using Horvath’s pan-tissue clock. Robust regressions examined overall and sex-specific associations. Higher prenatal DEHP exposure (*B* = 6.52, *95% CI* = 1.22, 11.81) and increased EAA (*B* = 2.98, *95% CI* = 1.64, 4.32) independently predicted more URIs. In sex-specific analyses, some similar effects were noted for boys, and EAA mediated the association between prenatal DEHP exposure and URIs. In girls, higher prenatal DEHP exposure was associated with decreased EAA, and no mediation was noted. Higher prenatal DEHP exposure may be associated with increased susceptibility to early childhood URIs, particularly in boys, and aging biomarkers such as EAA may be a biological mechanism. Larger cohort studies examining the potential developmental immunotoxicity of phthalates are needed.

## 1. Introduction

An upper respiratory infection (URI), also known as the ‘common cold’, is an infection of the nose, sinuses, or throat that can occur year round [1]. URIs are a heterogenous group of infections caused by viruses belonging to several different families, with the most common viral pathogens being rhinoviruses, influenzas A and B, parainfluenza virus, respiratory syncytial virus (RSV), and coronaviruses [2]. Before the age of two, infants and toddlers generally get six to eight colds each year, but some children may experience 10 or more each year [3]. Although there is variation in the symptoms (e.g., chills, nasal congestion, runny nose, coughing, sore throat) and duration of URIs (e.g., one to three weeks), frequent and persistent URIs in young children can indicate an increased susceptibility to infection related to atopic sensitization and subsequent development of atopic diseases such as asthma [4,5]. The increasing prevalence of both acute respiratory illnesses [6] and atopic diseases in children, particularly in those under five years of age [7,8], has prompted researchers to explore environmental factors that may be contributing to this trend [9,10]. It has been recommended that research is needed to investigate whether prenatal exposure to environmental chemicals may be linked to the frequency of early childhood URIs [11,12].

Phthalates are one class of environmental chemicals that may affect susceptibility to URIs and the later development of atopic diseases in children [11,13,14]. They are endocrine-disrupting chemicals (EDCs), and early exposure to phthalates has the potential to alter health and developmental trajectories [15]. One of the most prevalent phthalates is di(2-ethylhexyl) phthalate (DEHP). Exposure to DEHP occurs mainly through ingestion of contaminated food and drinking water, as well as dermal contact with polyvinyl chloride (PVC) plastic products such as vinyl flooring and children’s toys [16,17]. DEHP is commonly incorporated into PVC products in amounts from 40 to 65% and was once thought to have “no known adverse or toxic effects” [18], but increasing epidemiological evidence reports that DEHP is associated with negative health effects, including respiratory system complications [19]. Prenatal exposure to DEHP is of particular concern, as phthalates can transfer to the developing fetus across the placenta, are detectable in placental tissue, amniotic fluid, umbilical cord blood, and meconium, and could have adverse developmental effects on the fetus [20,21,22,23,24]. Based on findings from animal models, prenatal exposure to DEHP has been hypothesized to enhance susceptibility to infections by weakening innate immunity [25,26] and, thus, may contribute to the developmental origins of atopic diseases [27].

The potential biological mechanisms through which prenatal exposure to phthalates may contribute to immunity and infection are not well understood. Some studies in mice have reported that DEHP exposure can increase levels of immunological biomarkers (e.g., immunoglobulin G1 (IgG1), immunoglobulin E (IgE), interleukin 4 (IL4)) and induce airway inflammation [28,29]. However, no consistent pattern of results has emerged from murine research investigating the effects of phthalates on immune system function; an earlier review noted that studies had reported immunopotentiation, immunosuppression, and even null effects [13]. In a recent review of the potential immunotoxicity of phthalates in model organisms, higher DEHP exposure was reported to inhibit immune system function and interfere with immune signaling pathways, with some research indicating that these changes were mediated by DEHP’s effects on gene expression and regulation [30]. Further, DEHP has also been purported to affect other innate immune pathways (e.g., macrophage response) in animal models of disease [31].

Several studies have examined prenatal exposure to DEHP and early immune system function in humans. In studies investigating prenatal exposure to phthalates and immune system indices in umbilical cord blood, prenatal DEHP was not associated with elevated immune biomarkers (i.e., IgE, thymic stromal lymphopoietin (TSL), interleukin-33 (IL-33)) [32,33] or altered immune cell profiles (e.g., cytokines, chemokines) [34]. Further, little is known about the potential effects of prenatal DEHP exposure on childhood susceptibility to respiratory illnesses. A study in the INMA (Infancia y Medio Ambiente; Environment and Childhood) birth cohort from Spain reported that maternal average exposure to DEHP (median concentration = 101.7 μg/g creatinine) during the first and third trimesters of pregnancy was associated with higher odds of children developing wheeze, respiratory tract infections, bronchitis, and asthma from birth until seven years of age; although, the association with respiratory infections was attenuated in adjusted models [11]. In another study in the Odense Child Cohort from Denmark, prenatal DEHP exposure was not significantly associated with risk of the URI symptom of rhinitis in children at age five; although, a trend between higher exposure and greater risk was noted [35]. Given the documented increased susceptibility to respiratory tract infections in children with atopic diseases such as asthma [36,37] and research indicating that DEHP exposure likely plays a role in innate immune response to respiratory infections [26,38], more research is needed that examines the associations between prenatal exposure to DEHP and the frequency of early childhood URIs, as well as potential biological mediators of these associations.

DNA methylation is a plausible biomolecular mediator of the associations between prenatal DEHP exposure and health outcomes, such as susceptibility to URIs. DNA methylation is a stable and mitotically heritable epigenetic marker that can regulate gene expression, and as such, it is ideal for studying the potential biological embedding of early life chemical exposures with later life health [39]. There has been a call for investigations of the associations between early phthalate exposure and epigenetic mechanisms [40,41], particularly using novel tools such as epigenetic clocks (i.e., clocks that use algorithms to calculate the age of tissues based on DNA methylation) [42]. Epigenetic clocks are accurate predictors of DNA methylation-based age, also known as biological age [43]. These epigenetic biomarkers can provide insights into how environmental chemical exposures may affect biological aging and be examined as potential biological mediators. While most epigenetic clocks were developed using adult populations, several perform well in pediatric populations. For example, the Horvath pan-tissue clock [44] accurately estimates DNA methylation age across the lifespan in most human tissues. Epigenetic age acceleration (EAA) as estimated by the Horvath clock has been robustly associated with both early life exposures and later life morbidity and mortality [45], including respiratory illness outcomes [46]. EAA has also been consistently associated with, and even hailed as an emerging biomarker of asthma and atopic diseases in children [47,48] and may provide insight into the biological embedding of early life chemical exposures on immune system development. For example, one study found that accelerated epigenetic age in children was associated both with an increase in allergy and serum IgE [47], an antibody involved in allergic response mechanisms, which has also previously been found to increase with higher prenatal exposure to phthalates [32].

Due to the endocrine-disrupting nature of phthalates, DEHP could have sex-specific effects on epigenetic age. This is supported by a recent study, which reported that higher prenatal DEHP was associated with decreased epigenetic age acceleration (as calculated using the Horvath pan-tissue clock) in males, but not females, at seven years of age [42]. However, to our knowledge, no prior research has examined prenatal DEHP exposure and epigenetic aging rates in infants, or whether EAA may mediate associations between prenatal DEHP exposure and early childhood immune health outcomes, such as susceptibility to infections. Based on the previous literature, it is hypothesized that higher prenatal exposure to DEHP would be associated with a greater number of URIs in early childhood, biological aging would mediate this association, and associations would be sex-specific. To help address the current knowledge gaps, the present study examined the overall and sex-specific associations between prenatal exposure to DEHP, DNA methylation-based aging (i.e., EAA), and early childhood frequency of URIs in a sample of 69 maternal-child pairs from a Canadian pregnancy cohort.

## 2. Results

### 2.1. Sample Characteristics

Maternal and child characteristics for the overall sample (Table 1) and sex-stratified groups are presented (Tables S1 and S2). The participant flowchart is also provided (Figure S1). In the overall sample, mothers were mainly university-educated (73.91%), born in Canada (89.86), and had a median household income of >CAD 70,000 (CAD70k) (81.16%); all mothers were married/cohobating (100.00%). For just over half of the women, this was their first pregnancy (52.17% = primiparous). Children (49.28% female) were mainly white (92.75%), born between 35 and 42 weeks of gestation (M = 39.49, SD = 1.40 weeks), and weighed between 2260 and 4904 g (M = 3479.42, SD = 505.91 g). Sample characteristics did not differ substantially between the groups stratified by child sex (n = 34 girls and n = 35 boys).

### 2.2. Phthalate Exposure

Detection rates, minimums, maximums, geometric means (GMs), and percentiles for the individual DEHP metabolites (μg/g creatinine), mono(2-ethylhexyl) phthalate (MEHP), mono(2-ethyl-5-hydroxy-hexyl) phthalate (MEHHP), mono(2-ethyl-5-oxohexyl) phthalate (MEOHP), and mono(2-ethyl-5-carboxypentyl) phthalate (MECPP), and the molar sum of these four metabolites (μmol/g creatinine) are reported (to three significant figures) for the overall sample population (Table 2) and sex-stratified groups (Table S3). All metabolites were above the limit of detection (LOD) in 100% of maternal second trimester urine samples. Of the metabolites, MECPP had the highest GM (16.6 μg/g creatinine in the overall sample; 16.7 and 16.6 μg/g creatinine in girls and boys, respectively), and the molar sum of DEHP (herein referred to as DEHP)had a GM of 0.0814 μmol/g creatinine in the overall sample and similar levels in each sub-group (0.0824 and 0.0805 μmol/g creatinine in girls and boys, respectively).

### 2.3. Estimated Cell Type Proportions

Estimated cell type proportions were within the expected ranges for infants who were approximately three-months of age [49]. Preparatory analyses showed that across all 12 estimated cell type proportions, there were no significant associations with either prenatal DEHP exposure (Figure S2) or childhood URIs (Figure S3) within the overall sample. There were also no significant associations in sex-stratified analyses. In girls, cell type proportions were not associated with prenatal DEHP (Figure S4) or childhood URIs (Figure S5). In boys, cell type proportions were also not associated with prenatal DEHP (Figure S6) or childhood URIs (Figure S7).

### 2.4. Epigenetic Age Acceleration

Descriptive analysis of EAA showed that it was normally distributed and centered around zero (Figure S8). As the age range for the sample collection was narrow (i.e., M = 12.5 weeks, SD = 1.0 week), EAA (i.e., derived from the residuals of a regression of chronological age on predicted biological age) and epigenetic age difference (i.e., derived from the difference of chronological age minus predicted biological age) were identical (Figure S9).

### 2.5. Overall Associations between Prenatal DEHP Exposure, Epigenetic Age, and Early Childhood URIs

**Figure 1:**
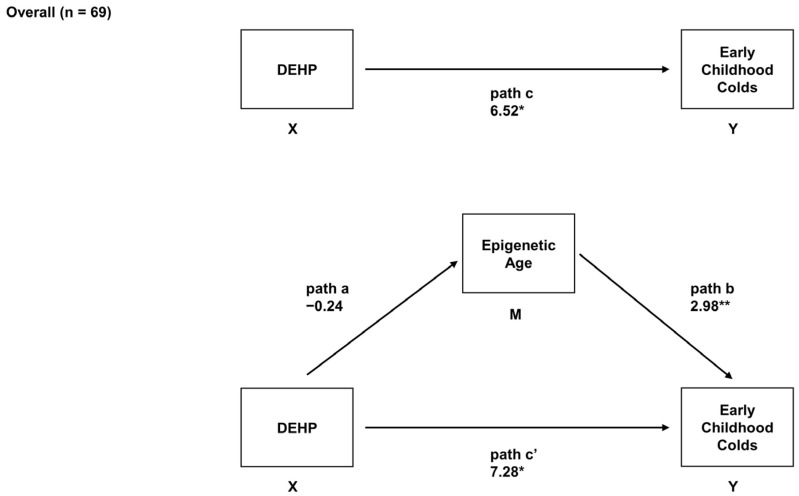
Mediation model in which infant EAA was examined as a mediator of prenatal exposure to DEHP and the number of early childhood colds in the overall sample (n = 69). This model depicts the relation of prenatal DEHP exposure to early childhood colds (path c; total effect), the relation of prenatal DEHP exposure to EAA (path a), the relation of EAA to early childhood colds adjusted for prenatal DEHP exposure (path b), and the relation of prenatal DEHP exposure to early childhood colds adjusted for EAA (path c’; direct effect). Unstandardized regression coefficients and *95% CIs* are reported. ** adjusted *p* < 0.05. * adjusted *p* < 0.10.

In the main analyses, adjusted robust regression models were used to determine the associations between prenatal DEHP, EAA, and children’s number of early childhood URIs (i.e., colds). In the overall sample, higher prenatal exposure to DEHP was associated with a greater number of early childhood colds (*B* = 6.52, *95% CI* = 1.22, 11.81, adjusted *p* = 0.07). Prenatal DEHP exposure was not associated with EAA (*B* = −0.24, *95% CI* = −1.24, 0.76, adjusted *p* = 0.63), but increased EAA was associated with a greater number of early childhood colds in the overall sample (*B* = 2.98, *95% CI* = 1.64, 4.32, adjusted *p* = 0.001). No evidence of mediation was found in the overall sample (indirect effect = −1.40, *95% CI* = −3.94, 1.65, adjusted *p* = 0.37) (Figure 1).

### 2.6. Sex-Specific Associations between Prenatal DEHP Exposure, Epigenetic Age, and Early Childhood URIs

**Figure 2:**
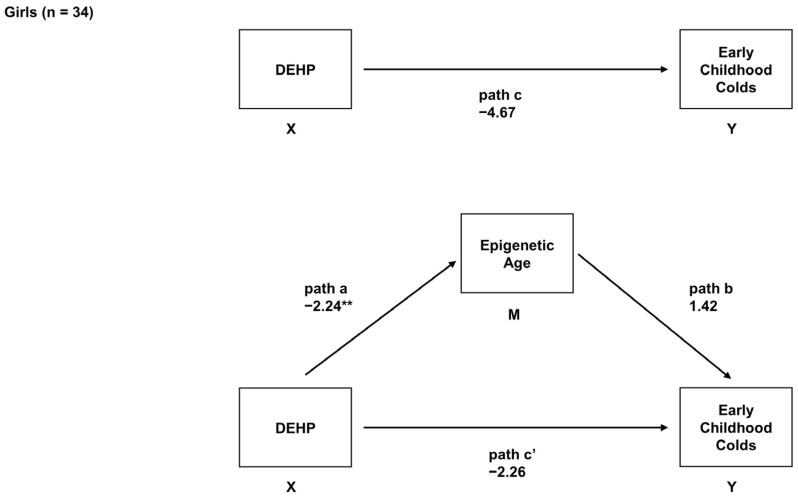
Mediation model in which infant EAA was examined as a mediator of prenatal exposure to DEHP and the number of early childhood colds in girls only (n = 34). For this sub-sample, this model depicts the relation of prenatal DEHP exposure to early childhood colds (path c; total effect), the relation of prenatal DEHP exposure to EAA (path a), the relation of EAA to early childhood colds adjusted for prenatal DEHP exposure (path b), and the relation of prenatal DEHP exposure to early childhood colds adjusted for EAA (path c’; direct effect). Unstandardized regression coefficients and *95% CIs* are reported. ** adjusted *p* < 0.05.

In the main analyses, adjusted robust regressions were also used to determine the associations between prenatal DEHP, EAA, and children’s number of colds in sex-stratified models. In girls, prenatal exposure to DEHP was unrelated to the number of early childhood colds (*B* = −4.67, *95% CI* = −13.33, 3.99, adjusted *p* = 0.28). Prenatal DEHP exposure was associated with decreased EAA (*B* = −2.24, *95% CI* = −3.43, −1.05, adjusted *p* = 0.001), but EAA was not associated with the number of early childhood colds in girls (*B* = 1.42, *95% CI* = −1.38, 4.22, adjusted *p* = 0.41). No evidence of mediation was found in girls (indirect effect = −3.32, *95% CI* = −8.76, 2.22, adjusted *p* = 0.22) (Figure 2).

**Figure 3:**
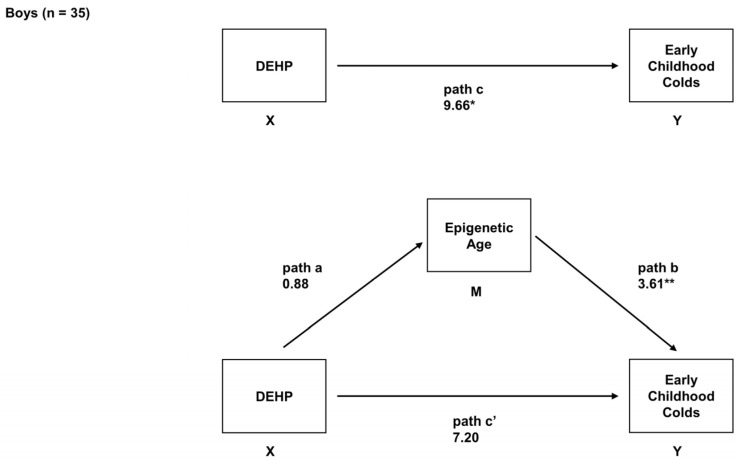
Mediation model in which infant EAA was examined as a mediator of prenatal exposure to DEHP and number of early childhood URIs colds in boys only (n = 35). For this sub-sample, this model depicts the relation of prenatal DEHP exposure to early childhood colds (path c; total effect), the relation of prenatal DEHP exposure to EAA (path a), the relation of EAA to early childhood colds adjusted for prenatal DEHP exposure (path b), and the relation of prenatal DEHP exposure to early childhood colds adjusted for EAA (path c’; direct effect). Unstandardized regression coefficients and *95% CIs* are reported. ** adjusted *p* < 0.05. * adjusted *p* < 0.10.

In boys, higher prenatal exposure to DEHP was associated with a greater number of early childhood colds (*B* = 9.66, *95% CI* = 2.39, 16.94, adjusted *p* = 0.05). Prenatal DEHP exposure was not associated with EAA (*B* = 0.88, *95% CI* = −0.55, 2.31, adjusted *p* = 0.22), but increased EAA was associated with a greater number of early childhood colds in boys (*B* = 3.61, *95% CI* = 1.64, 5.58, adjusted *p* = 0.001). We found evidence of mediation (indirect effect = 3.26, *95% CI* = 1.80, 4.51, adjusted *p* = 0.09); specifically, EAA mediated the association between prenatal DEHP and the number of early childhood colds in boys (Figure 3).

### 2.7. Post Hoc and Sensitivity Analyses

*Post hoc* analyses examined the associations between prenatal DEHP, other biological aging predictions, and children’s number of colds. The direction and magnitude of effects were similar in models that examined associations with intrinsic epigenetic age acceleration (IEAA), which was EAA corrected for estimated cell type proportions [44] (Table 3). In the analyses, which examined associations with EAA estimated using a different epigenetic clock tool that is tissue-appropriate, the Horvath skin and blood clock, which is less accurate in pediatric samples than the Horvath pan-tissue clock [50], a mediating effect was noted in boys (indirect effect = 3.15, *95% CI* = 0.04, 9.52) and in girls (indirect effect = −3.90, *95% CI* = −10.56 − 0.23) (Table 3). Estimates were largely unchanged in the sensitivity analysis that adjusted for prenatal exposure to bisphenol A (BPA) (Table S4).

## 3. Discussion

In this prospective study using data from the Alberta Pregnancy Outcomes and Nutrition (APrON) cohort, we observed that higher maternal second trimester urinary DEHP concentrations and increased epigenetic age acceleration (EAA) were independently associated with a greater number of early childhood upper respiratory infections (URIs). Further, our analyses stratified by child sex revealed several unique associations. In girls, higher prenatal exposure to DEHP was associated with decreased EAA (i.e., slower biological aging). While, in boys, we found some similar effects as those noted in the overall models (i.e., positive total effect and positive path b), but the magnitude of these effects was larger in boys. Also in boys, we found an indirect effect; EAA mediated the association between prenatal exposure to DEHP and early childhood URIs. To our knowledge, no prior research has examined the association between prenatal exposure to DEHP and epigenetic aging rates in infants, nor whether EAA may mediate associations between prenatal DEHP and immune health outcomes. The present analyses were exploratory and limited by the small sample size. Although these findings require replication, this study provides novel evidence suggesting that prenatal exposure to DEHP may increase susceptibility to the ‘common cold’ in young children and that EAA may be a potential biological mechanism, which transmits risk for viral infections.

The common cold is the most frequent infection experienced in childhood and poses an enormous economic burden in terms of healthcare utilization, pediatric consultations, and absences from childcare, school, and the parental workplace [51,52]. We found that higher prenatal exposure to DEHP was associated with a greater number of early childhood colds, particularly in boys (*B*’s 6.52 and 9.66 in the overall sample and boys, respectively). This finding suggests that in utero exposure to DEHP may exert immunosuppressive effects in children, with potentially more adverse effects in boys. Immune-related effects have been documented following phthalate exposure in animal models; although results are mixed, predominantly immunosuppressive effects are reported [13]. Similarly, phthalate exposure is acknowledged to affect human respiratory health and is thought to play a role in susceptibility to respiratory infections, but the overall evidence is weak and inconsistent [53]. To our knowledge, no prior work has linked prenatal exposure to phthalates to increased incidence of the common cold. A nationally representative cross-sectional study from the United States reported that exposure to other endocrine-disrupting chemicals (EDCs), per- and polyfluoroalkyl substances (PFAS), was associated with greater odds of developing colds in children and adolescents, with larger effects in boys [54]. It is possible that prenatal exposure to endocrine disruptors could alter immune system development and susceptibility to infectious diseases in young children by disrupting the activity of sex steroid hormones [30,55]. Toxicology research indicates that DEHP and its metabolites disrupt androgen signaling pathways and can exert anti-androgenic effects [56,57]. In animal models, prenatal DEHP exposure has been shown to reduce androgen (e.g., testosterone) activity in pregnant dams and male offspring [58,59]. In humans, higher exposure to DEHP has been associated with lower serum testosterone concentrations in pregnant women, regardless of fetal sex [60], as well as in cord blood [61]. Generally, androgens such as testosterone have been shown to demonstrate immunosuppressive effects [55], but the effects of in utero exposure to DEHP on androgen levels in early development and the implications for children’s immune system function are still unknown. Given the significant societal and economic burden associated with the common cold, more research into the underlying mechanisms that transmit risk for respiratory illnesses following early exposure to environmental chemicals is needed. As a male bias is often observed in infectious disease incidence [62], further research examining the role of sex in immune function is strongly recommended.

The possible immune-related dysfunctions that may confer susceptibility to infection following prenatal exposure to DEHP, potentially in a sex-specific manner, are not well understood. In model organisms, DEHP has been shown to contribute to the decline of immune functions through increasing reactive oxygen species (ROS), interfering with the expression of immune genes (e.g., *TSLP*, *TSLPR*, *IL-7R*) and affecting immune cells (e.g., T cells, BK cells) and cytokines [30]. The mechanisms by which prenatal exposure to phthalates interferes with immune cells and immune system function in young girls and boys remains unclear. The Hokkaido Study on Environment and Children’s Health from Japan found that maternal second trimester concentrations of one DEHP metabolite, mono(2-ethylhexyl) phthalate (MEHP), were associated with lower cord blood immunoglobin E (IgE) levels in overall models, but not in sex-stratified analyses [63]. Conversely, both the Maternal-Infant Research on Environmental Chemicals (MIREC) study from Canada and Barwon Infant Study (BIS) from Australia did not find associations between prenatal exposure to DEHP and umbilical cord blood immune system biomarkers in overall or sex-stratified analyses [32] or models adjusted for child sex [34], respectively. Notably, we also did not find any associations with DEHP exposure and altered proportions of immune cell types. However, the MIREC study found that maternal urinary mono(3-carboxypropyl) phthalate (MCPP) concentrations during pregnancy were associated with increased levels of IgE and interleukin-33 (IL-33)/ thymic stromal lymphopoietin (TSLP) in their overall, but not their sex-stratified, analyses [32]. Similarly, other phthalates (e.g., di-n-butyl phthalate (DnBP), molar sum of dibutyl phthalates (DBPs), and benzyl butyl phthalate (BBzP)) have been linked to altered immune cell (e.g., cytokines, chemokines) profiles in cord blood [33,34]. These altered immune profiles are likely related to infection susceptibility [33] and are thought to explain the role of prenatal exposure to phthalates in the etiology of childhood atopy [32,63]. Although the current examination of prenatal DEHP exposure and childhood colds (i.e., a measure related to immune health) is not directly comparable to these other examinations of immunological profiles, this evidence cumulatively suggests that prenatal phthalate exposure may have immunotoxic effects.

One of the proposed modes of action of DEHP during pregnancy that contributes to adverse health effects is through activation of the peroxisome proliferator activated receptor (PPAR) gamma (γ) signaling pathway [64]. This is a potential pathway through which prenatal exposure to DEHP may affect children’s immune system development. For example, the DEHP metabolite MEHP has been shown to inhibit and induce apoptosis of immune cells (i.e., B cells, which produce antibodies to protect from infection) in bone marrow, which is thought to occur through activation of PPARγ [65]. Similarly, a recent study indicated that activation of PPAR alpha (α) and PPARγ by DEHP and MEHP suppresses macrophage (i.e., white blood cells whose primary function is to phagocytize foreign particles such as viruses) activity in the liver [66]. A recent review acknowledged that phthalates likely cause dysregulation of the immune system through diverse mechanisms, including PPARγ, reduced biosynthesis of testosterone, and aryl hydrocarbon receptor (AHR) dysregulation [67]. Thus, given the mixed evidence of cell type and immune marker differences in blood, and the relevance of DEHP exposure for mast cell maturation and macrophage activity, it is possible that other biological samples, rather than blood, may be more appropriate for direct examination of phthalate immunotoxicity in future studies [31,67]. It is tempting to hypothesize that EAA, a cumulative biomarker, was able to detect perturbation in immune function. Although our preparatory analyses did not show that the blood cell proportions accounted for this association, it is possible that EAA may be able to detect differences in immune cell maturation or function due to DEHP exposure. More research is needed to broadly examine the potential immunotoxicity of prenatal phthalates to better understand if they impair innate immune responses [25,26] in a sexually dimorphic manner [68] and whether they contribute to the developmental origins of respiratory and atopic diseases [27].

A central focus of this study was the investigation of the influence of an environmental factor, prenatal exposure to phthalates, on EAA, as it related to a relevant clinical phenotype, early childhood colds. We found thought-provoking associations between epigenetic aging rates in infants and the number of colds from 12 to 18 months of age in the overall sample and in boys; we found that increased epigenetic age acceleration was associated with a greater number of early childhood colds (*B*’s 2.98 and 3.61 in the overall sample and boys, respectively). In girls, no evidence of an association between EAA and early childhood URIs was found (i.e., adjusted *p* > 0.10). Previously, DNA methylation alterations have been linked to susceptibility to URIs [69,70], and our findings may elucidate epigenetic aging as a potential DNA methylation-based indicator of infection risk in young children, but additional research is needed to replicate and clarify these sex-specific findings. Epigenetic age deviations in children are likely associated with developmental trajectories, diseases, and certain environmental conditions that may accelerate or decelerate biological development in early life and childhood [71]. Notably, many epigenetic clocks were developed for use with adult populations and have been found to not accurately predict the age of individuals under the age of 20 [43]. The Horvath pan-tissue clock is recommended for pediatric blood samples [50], and positive EAA estimated using this clock has been previously associated with greater odds of atopic sensitization in children from Project Viva [47]. At this time, more work is required to better understand whether pediatric epigenetic age estimates are a driving force or the result of specific pediatric phenotypes [71]. After it has been clarified whether epigenetic age estimates are a driving force or resultant from phenotypes how increased epigenetic age acceleration relates to susceptibility to infection and the experience of early life immune events can be further interpreted.

The developmental origins of health and disease (DOHaD) conceptual model suggests that epigenetic modifications, such as DNA methylation alterations, are a mechanism through which early environmental exposures transmit risk for later health and disease [72]. There has been a call for investigations of early phthalate exposure on DNA methylation profiles [40,41], particularly in infants and young children. We found that higher prenatal exposure to DEHP was associated with slower biological aging, or DNA methylation-predicted age that was ‘younger’ than chronological age in infant girls. This finding is intriguing, as accelerated epigenetic age is typically associated with greater morbidity and mortality in adult populations [73,74], but what ‘faster’ or ‘slower’ biological aging means in pediatric populations is uncertain. In children, it is possible that any deviation from concordance between biological and chronological age—regardless of faster or slower—could be indicative of environmental experiences that may influence future health and development. Potentially, decreased EAA in young children may reflect developmental deceleration or reflect a developmentally younger pattern of DNA methylation [75]. The current understanding of the biological implications of EAA in pediatric populations remains inconclusive and may very well be situationally dependent. The present findings provide additional support for the contention that an epigenetic biomarker of prenatal exposure to phthalates may be evident in infants as young as three months of age [76], and indicates that epigenetic markers of biological aging, EAA, in young infants are sensitive to prenatal exposure to DEHP. This may be influenced by sex differences in aging, as men have been found to display positive EAA compared to women, across epigenetic clocks (e.g., Horvath pan-tissue, Horvath skin and blood, Hannum, GrimAge, PhenoAge, and Pace of Aging) [77,78]. Additional investigations examining associations between prenatal phthalate exposures, as well as other environmental chemicals (e.g., metals, air pollutants, pesticides), and epigenetic clocks in very young pediatric samples may provide novel insights into the early molecular programming of disease susceptibility and contribute to the continued evolution of the DOHaD paradigm [79].

Given the potential sensitivity of the developing immune system to early phthalate exposure, researchers have called for critical policy reforms and stricter regulations on phthalates such as DEHP to protect healthy development in children. The limited evidence on potential biological mechanisms through which phthalates may adversely affect immune system development may be one of the reasons that these chemicals are still considered safe exposures for pregnant women and children [80,81]. This was the first study to examine infant epigenetic age as a potential biomolecular mediator of the immunosuppressive effects of prenatal exposure to DEHP, and future well-powered investigations are needed to replicate and validate the present findings. Based on results from animal models, DNA methylation alterations are hypothesized to underlie the associations between exposure to phthalates and adverse health outcomes in human populations [40,82], including susceptibility to viral infections [83]. We found support for these hypothesized associations in our mediation analyses. Specifically, we found that EAA mediated the association between prenatal DEHP exposure and the number of early childhood colds in boys (indirect effect = 3.26); this finding should be cautiously interpreted. In our supplementary analyses, which examined other tissue-appropriate epigenetic age indices, we also found an indication that EAA (estimated from the Horvath skin and blood clock) mediated this association in boys and girls (indirect effects 3.15 and −3.90 for boys and girls, respectively). These findings suggest that biological aging may be a factor underlying the association between prenatal DEHP exposure and children’s development of upper respiratory illnesses and supports the examination of epigenetic age as a potential mediator of child health outcomes in future investigations. A recent epigenome-wide investigation indicated that prenatal exposure to phthalates may program the activity and signaling pathways of immune cells and sex steroid hormones in infants [76], both of which have important implications for sex differences in the capacity to cope with infections and the later development of inflammatory conditions and autoimmune disorders [84,85]. Additional work is needed to understand how prenatal DEHP exposure contributes to developmental immunotoxicity [80,81] and the biological (e.g., immunological, epigenetic) mechanisms that transmit risk in young girls and boys.

Our statistical confidence in these preliminary findings is strengthened by our analytical approach, which included the use of robust models and correction for multiple comparisons. However, the small sample size, especially given our adjustment for relevant covariates and the smaller sub-samples used for the sex-stratified analyses, is a notable limitation. Our main analyses examined DNA methylation-based age, as predicted by the Horvath pan-tissue clock [44], which is the most widely-accepted clock for estimating epigenetic aging in all age groups, including pediatric populations [71]. We also found similar effect estimates in our *post hoc* analyses, which used other epigenetic age predictions, and our sensitivity analysis, which adjusted for prenatal exposure to BPA. Our outcome measure, the number of early childhood colds, was obtained from maternal reports and is subject to recall bias. It is possible that unmeasured confounders biased effect estimates. Phthalate monoesters are detectable in human breastmilk, and their concentrations have been found to correlate with important predictors of later immune system outcomes such as hormone concentrations in infants [86]. It is unclear how maternal DEHP exposure may influence breastmilk composition, but it could impact the endocrine control of lactation, thereby disturbing the immune components in breast milk and impacting infant immune system development and response to infections [87]. It is also possible that maternal exposure to DEHP may influence maternal susceptibility to infections and as a result, impact the frequency of URIs in infants. This study did not have data available on maternal breast milk composition or maternal susceptibility to URIs, and these factors should be examined in future research. This was an exploratory examination and additional well-powered studies (e.g., larger cohort studies) are needed to clarify the associations between prenatal phthalate exposures, epigenetic biomarkers, and children’s susceptibility to infection.

## 4. Materials and Methods

### 4.1. Participants and Procedure

Participants included a sample of maternal–child pairs (n = 69) recruited between 2009 and 2012 from the APrON study [88,89]. Inclusion criteria for the present study were as follows: (i) a maternal spot urine sample provided during the second trimester of pregnancy, (ii) mothers did not report smoking, consuming alcohol, or receiving steroids during pregnancy, (iii) a venous blood sample obtained from infants at three-months of age, and (iv) data available on the number of colds children had from 12- to 18-months of age (see Figure S1 for participant flowchart). The research protocol was approved by the Conjoint Health Research Ethics Board at the University of Calgary (Ethics ID: REB14-1702). Written, informed consent was obtained from families prior to the collection of biospecimens and the completion of questionnaires.

### 4.2. Urinary DEHP Assessment

Maternal spot urine samples were collected during the second trimester of pregnancy (M gestational age of 17.0 ± 2.1 weeks). The methods describing the urinary sample collection protocol, quality control experiments, and quantification of phthalate metabolites at the Alberta Centre for Toxicology have been previously described [90,91]. Four monoester phthalate metabolites of DEHP were quantified using liquid chromatography-tandem mass spectrometry (QTRAP 5500, AB Sciex, Concord, ON, Canada). The analytes included MEHP, MEHHP, MEOHP, and MECPP. The limit of detection (LOD) for all metabolites was 0.10 μg/L, and for statistical modeling, all values below the LOD were assigned the value of the LOD/√2 [92]. The molar sum of DEHP metabolites was also calculated [91,93]. Aliquots (1 mL) of the same urine samples were also analyzed quantitatively for creatinine at the Clinical Trials Laboratory, Alberta Health Services (Edmonton, AB, Canada). To account for urinary dilution, creatinine-adjusted concentrations of DEHP (μmol/g creatinine) were used in the analyses.

### 4.3. Children’s URIs

A sub-sample of APrON mothers and infants participated in an 18-month clinic visit [94]. During this visit, mothers completed a brief infant health and development questionnaire. One item from this questionnaire was used to determine children’s URIs for the present study: “How many colds has your child had in the last 6 months?” Mothers reported on the number of colds their child had in the 6-month (1/2 year) period from 12-months to 18-months of age (mean number of colds = 2.5, SD = 1.5, range: 1–7).

### 4.4. Epigenetic Age Calculation

#### 4.4.1. Infant Blood Sample Collection

Venous blood samples were collected when infants were three-months of age (M = 2.9, SD = 0.2) using established protocol [49,76]. Venipuncture blood draws were performed by a certified pediatric phlebotomist at the Alberta Children’s Hospital. Whole blood samples were collected and stored for less than 6 h at −80 °C prior to processing. To separate whole blood into plasma, buffy coat, and erythrocytes, samples were centrifuged at 3000 RCF for 15 min. This was completed to separate and aliquot the buffy coat preparation, which consists of peripheral blood mononuclear cells (PBMCs) and some granulocytes, though a much lower proportion of granulocytes than in whole blood preparations. The buffy coat samples were stored at −80 °C and shipped on dry ice to the Department of Medical Genetics at the University of British Columbia for DNA extraction and quantification.

#### 4.4.2. DNA Methylation Assay and Data Processing

DNA methylation data were extracted and processed using established protocol [49,76]. In brief, a sample of 750 ng of genomic DNA was used for bisulfite conversion using the EZ DNA Methylation Kit (Zymo Research, Irvine, CA, USA). Following the manufacturer’s protocol, quantification of DNA methylation status of purified bisulfite converted DNA was performed using the Infinium HumanMethylation450 (450k) Bead Chip Assay (Illumina, San Diego, CA, USA). This platform quantified DNA methylation status at >450,000 CpG sites; this covers 99% of available reference sequence genes [95]. A sample of 160 ng of bisulfite converted DNA was whole genome amplified, fragmented, and hybridized to the BeadChip arrays. Via the 450k platform, each CpG site is targeted by a probe to distinguish “methylated” and “unmethylated” intensity through different dye colors (i.e., green and red). Methylation levels, also known as beta (β) values, were calculated. This was completed in Illumina GenomeStudio (San Diego, CA, USA) by dividing the methylation probe signal intensity by the sum of the methylated and unmethylated probe signal intensities). The β values range from 0 (i.e., completely unmethylated site) to 1 (i.e., fully methylated site). The β values were extracted and imported into R statistical software for processing and analysis. Further detail on the 450k arrays for the APrON cohort has been previously published [76]. The raw data were pre-processed through the same pipeline as previously used [49,76]; this study used the pre-processed DNA methylation data to calculate epigenetic age.

#### 4.4.3. Estimated Cell Type Proportions

Defining cell type identity is a major biological function of DNA methylation, and as such, proportions of immune cells making up blood can be defined using DNA methylation profiles. Therefore, cell type proportions in buffy coat blood were estimated using constraint projection [96] on the IDOL (Identifying Optimal Libraries) extended cell type reference data set and version 1.8.0 of the R package *FlowSorted.BloodExtended.EPIC* [97,98,99] to derive 12 cell type proportions: neutrophils, eosinophils, basophils, naïve and memory B cells, natural killer (NK) cells, naïve and memory CD4-T cells, T regulatory cells, naïve and memory CD8-T cells, and monocytes. Our samples were from young infant blood. Thus, based on the approach previously used [49,100], available cord blood references for deconvolution were not used, as these references account for nucleated red blood cells (NRBCs; immature blood cells), a cell type not found in the blood of infants at three-months of age [101].

#### 4.4.4. Epigenetic Clock

In the main analysis, epigenetic age, or biological age, as determined by the pattern of β values in the methylome, was calculated using the Horvath pan-tissue clock, and in the *post hoc* analysis using the Horvath skin and blood clock, using the online age calculator: https://dnamage.genetics.ucla.edu/ (accessed on 22 February 2020) [44]. These measures are appropriately applied to venous blood and have been validated and employed frequently in research [102], as well as in similarly aged children [102,103]. A recent review concluded that the Horvath pan-tissue clock, a widely used age estimator based on DNA methylation at 353 CpG sites developed using DNA methylation data from over 8000 samples, was the most accurate in pediatric blood samples [50]. A normalized, background- and color-corrected β file was provided for the calculator. Epigenetic age acceleration (EAA) was defined as the residuals from regressing the calculated epigenetic age on the infants’ actual chronological age. This provides a measure of whether infants are aging faster (increased EAA) or slower (decreased EAA) biologically than their chronological age; this was the main biological aging estimate used in the present analyses.

Other biological aging predictions were also calculated and compared with EAA. Epigenetic age difference was calculated as the estimated epigenetic age subtracted from the reported chronological age. The intrinsic epigenetic age acceleration (IEAA) measure was outputted by the calculator as residuals from the regression of biological age on chronological age, accounting for reference-estimated cell type proportions [44]. Lastly, in a *post hoc* analysis, EAA was calculated using the Horvath skin and blood clock to attempt to replicate our findings in this same sample with a tool employing a unique set of 391 CpGs [104].

### 4.5. Covariates

Based on variables previously identified to be associated with prenatal phthalate exposure, epigenetic modifications, and/or children’s colds [11,76,105], this study considered several covariates. Potential covariates included the following maternal characteristics: age, education, household income, self-reported race, parity, history of asthma, and pre-pregnancy body mass index (BMI). Potential covariates also included the following child characteristics: gestational age at birth, birthweight, month of birth, and childcare arrangements at 12-months of age. Dichotomous variables were coded as follows: household income (i.e., <$70,000 CAD versus >$70,000 CAD), maternal education (i.e., undergraduate university degree or higher versus trade school/high school diploma/lower), maternal self-reported race (i.e., White versus not White), maternal parity (i.e., primiparous versus multiparous), maternal history of asthma (i.e., yes versus no), and childcare arrangements (i.e., childcare utilized versus not utilized). All mothers (n = 69) reported breastfeeding at three-months postpartum; this was not examined as a covariate.

### 4.6. Statistical Analyses

All analyses were performed in R (version 4.3.1). To describe study sample characteristics, means (i.e., geometric means for phthalate metabolites, otherwise arithmetic means are reported) and standard deviations (SD) for continuous variables and proportions for categorical variables were used.

Preparatory analyses were conducted to describe and characterize the estimated cell type proportions and EAA. The associations between estimated cell type proportions and DEHP and URIs were examined using linear regressions with Bonferroni-corrected *p*-value significance threshold of *p* < 0.004.

The main analyses involved examining the associations between prenatal exposure to DEHP, EAA, and URIs. This was completed utilizing robust multivariable regressions. These regressions were completed using the R package *MASS* and with Huber M-estimation [106,107]. Overall models (n = 69) and models stratified by child sex (n = 34 girls; n = 35 boys) were examined. Covariates were included in final robust models if they were associated with the outcome at *p* < 0.20 [108]. Overall models were adjusted for three covariates (i.e., maternal self-reported race, maternal parity, and birth month), female-stratified models were adjusted for two covariates (i.e., maternal age and self-reported race), and male-stratified models were adjusted for four covariates (i.e., maternal education, maternal parity, maternal history of asthma, and child month of birth). Robust mediation tests were performed (using *ZYmediate* from the R package *WRS2*) using 10,000 bootstrapped samples and the method proposed by Zu and Yuan [109]. In regression approaches, mediating effects (i.e., indirect effects) can be examined even in the absence of other effects (e.g., direct effects, relation of X to M) [110]. Mediation analysis results are reported using the widely applied model notation [111]: relation of X to Y (path c; total effect), relation of X to M (path a), relation of M to Y adjusted for X (path b), relation of X to Y adjusted for M (path c’; direct effect), mediating effect (path ab; indirect effect). All tests were 2-sided. We corrected for multiple comparisons in the robust regression analyses using the Benjamini–Hochberg procedure to control the false discovery rate (FDR; i.e., proportion of false positives) [112]. Given the limited evidence on the effect of prenatal exposure to phthalates on early childhood EAA and immune health outcomes, we adopted an exploratory approach and report adjusted *p*-values (*q*-values) from 0.05 to 0.1. The adjusted *p*-value (*q*-value) thresholds of 0.05 and 0.10 yield FDRs of 5% and 10%, respectively. Thus, we can have the most statistical confidence in results at adjusted *p* < 0.05. Simulation analysis indicated that a sample size of 59 would be adequately powered (0.80) to detect a mediation effect, when using a percentile bootstrap method, and considering medium-to-large path a and b effects [113]. A larger sample size (≥78) would be required when considering smaller path a and b effects [113], and thus, these analyses are considered exploratory.

We did not examine EAA predictions from other epigenetic clocks in the main analyses, due to their poor performance in children [50]. However, *post hoc* analyses explored associations with (i) intrinsic epigenetic age acceleration (IEAA), which is corrected for cell types [44], and (ii) EAA estimated using Horvath’s skin and blood clock [104]. Sensitivity analysis also examined associations when prenatal exposure to another non-persistent endocrine disruptor, bisphenol A (BPA), was included as a covariate.

## 5. Conclusions

This exploratory study found that higher prenatal DEHP exposure and increased EAA were independently associated with a greater number of early childhood URIs, particularly in boys. Further, we found that EAA mediated the association between prenatal DEHP exposure and the number of URIs in boys. Lastly, we found that prenatal exposure to DEHP was associated with a decreased EAA in girls, possibly indicating developmental deceleration. As early childhood constitutes a window of susceptibility for the maturation and priming of the immune system, it is critical to examine the potential developmental immunotoxicity of phthalates and the sex-specific effects of these EDCs.

## Figures and Tables

**Table 1:** Maternal and child characteristics for the overall sample (n = 69).

	n (%)	Mean (SD)
**Maternal Characteristics**		
Age (years)	-	31.24 (4.01)
White	64 (92.75%)	-
Married/Cohabiting	69 (100.00%)	-
Household Income > CAD 70k ^1^	56 (81.16%)	-
**Child Characteristics**		
Birthweight (g)	-	3479.42 (505.91)
Gestational age at birth (weeks)	-	39.49 (1.40)
Sex (Female)	34 (49.28%)	-
Age at blood draw (weeks)	-	12.52 (0.96)
Number of colds	-	2.54 (1.51)

^1^ CAD (Canadian dollars).

**Table 2:** Creatinine-adjusted phthalate metabolite concentrations (μg/g creatinine) and DEHP (μmol/g creatinine) in maternal second trimester urines (n = 69).

Metabolite	% > LOD	Minimum	Maximum	GM	25th Percentile	50th Percentile	75th Percentile
MEHP ^1^	100%	0.712	31.0	3.52	2.26	3.12	4.95
MEHHP ^1^	100%	2.32	33.5	10.6	7.05	9.81	16.4
MEOHP ^1^	100%	2.58	25.7	9.13	6.53	8.83	13.0
MECPP ^1^	100%	6.41	62.4	16.6	11.9	16.0	23.3
DEHP ^2^	-	0.0222	0.285	0.0814	0.0534	0.0755	0.126

^1^ LOD = 0.10 μg/L.; ^2^ Molar sum of individual DEHP metabolites.

**Table 3:** *Post hoc* analyses which considered intrinsic epigenetic age acceleration (IEAA) and EAA estimated using Horvath’s skin and blood clock.

	IEAA	EAA (Skin and Blood Clock)
	B (95% CI)	B (95% CI)
**Overall Sample (n = 69)**		
Total Effect (path c) ^1^	-	-
Path a	−0.34 (−1.30, 0.61)	−0.03 (−0.58, 0.51)
Path b	2.98 ^†^ (1.67, 4.28)	4.15 ^†^ (1.38, 6.92)
Direct effect (path c’)	7.81 ^†^ (2.54, 13.07)	7.11 ^†^ (1.63, 12.58)
Indirect effect (path ab)	−1.79 (−5.41, 1.26)	−0.07 (−2.34, 2.35)
**Girls (n = 34)**		
Total Effect (path c) ^1^	-	-
Path a	−2.40 ^†^ (−3.54, −1.27)	−0.80 ^†^ (−1.59, −0.02)
Path b	1.04 (−1.88, 3.96)	3.47 (−0.79, 7.73)
Direct effect (path c’)	−3.47 (−15.03, 8.09)	−2.24 (−11.86, 7.39)
Indirect effect (path ab)	−2.11 (−8.56, 5.87)	−3.90 ^†^ (−10.56, −0.23)
**Boys (n = 35)**		
Total Effect (path c) ^1^	-	-
Path a	0.85 (−0.54, 2.25)	0.50 (−0.16, 1.17)
Path b	3.70 ^†^ (1.74, 5.66)	4.55 ^†^ (0.28, 8.81)
Direct effect (path c’)	7.29 (−0.12, 14.70)	8.38 (−0.11, 16.87)
Indirect effect (path ab)	3.27 (−0.78, 8.58)	3.15 ^†^ (0.04, 9.52)

^†^ Unadjusted *p* < 0.05. ^1^ Path not examined again as it is already reported in the results (Section 2.5 and Section 2.6).

## Data Availability

Data are contained within the article and Supplementary Materials.

## Supplementary Materials

The following supporting information can be downloaded at https://www.mdpi.com/article/10.3390/epigenomes8010003/s1. Figure S1. Flowchart of participants included in the present study; Figure S2. Estimated cell type proportions in three-month-old infant venous blood were not associated with prenatal DEHP exposure across the entire cohort (n = 69). Each graph represents the association between each of the 12 estimated cell type proportions (on the y-axis) and DEHP exposure (on the x-axis). On each graph is the Pearson’s correlation (*R*) and *p*-value of the correlation between estimated cell type proportion and DEHP exposure; Figure S3. Estimated cell type proportions in three-month-old infant venous blood were not associated with childhood upper respiratory infections (URIs) across the entire cohort (n = 69). Each graph represents the association between each of the 12 estimated cell type proportions (on the y-axis) and childhood URIs (on the x-axis). On each graph is the Pearson’s correlation (*R*) and *p*-value of the correlation between estimated cell type proportion and childhood URIs; Figure S4. Estimated cell type proportions in three-month-old infant girls’ venous blood were not associated with prenatal DEHP exposure (n = 34). Each graph represents the association between each of the 12 estimated cell type proportions (on the y-axis) and DEHP exposure (on the x-axis). On each graph is the Pearson’s correlation (*R*) and *p*-value of the correlation between estimated cell type proportion and DEHP exposure; Figure S5. Estimated cell type proportions in three-month-old infant girls’ venous blood were not associated with childhood upper respiratory infections (URIs) (n = 34). Each graph represents the association between each of the 12 estimated cell type proportions (on the y-axis) and childhood URIs (on the x-axis). On each graph is the Pearson’s correlation (*R*) and *p*-value of the correlation between estimated cell type proportion and childhood URIs; Figure S6. Estimated cell type proportions in three-month-old infant boys’ venous blood were not associated with prenatal DEHP exposure (n = 35). Each graph represents the association between each of the 12 estimated cell type proportions (on the y-axis) and DEHP exposure (on the x-axis). On each graph is the Pearson’s correlation (*R*) and *p*-value of the correlation between estimated cell type proportion and DEHP exposure; Figure S7. Estimated cell type proportions in three-month-old infant boys’ venous blood were not associated with childhood upper respiratory infections (URIs) (n = 35). Each graph represents the association between each of the 12 estimated cell type proportions (on the y-axis) and childhood URIs (on the x-axis). On each graph is the Pearson’s correlation (*R*) and *p*-value of the correlation between estimated cell type proportion and childhood URIs; Figure S8. Histogram with kernel density plot of epigenetic age acceleration (EAA) values estimated from the Horvath pan-tissue clock in these samples. The dashed line indicates the mean, which is centered at approximately zero, and the data are approximately normally distributed; Figure S9. Estimated epigenetic age acceleration (EAA) and epigenetic age difference from the Horvath pan-tissue clock were the same in this sample due to the narrow age range during blood sample collection. The Horvath pan-tissue EAA was derived from residuals of a regression of chronological age on predicted biological age is on the x-axis. While Horvath pan-tissue epigenetic age difference was derived from the difference of chronological age minus predicted biological age. As the correlation is so high, these measures of EAA are synonymous in this sample; Table S1. Maternal and child characteristics for the sub-sample stratified by child sex (n = 34 girls); Table S2. Maternal and child characteristics for the sub-sample stratified by child sex (n = 35 boys); Table S3. Creatinine-adjusted prenatal phthalate metabolite concentrations (μg/g creatinine) and DEHP (μmol/g creatinine) in maternal second trimester urine for the sex-stratified groups; Table S4. Sensitivity analysis adjusting for prenatal exposure to bisphenol A (BPA).
