# Supplementary material for: Sex-Specific Associations between Prenatal Exposure to Di(2-ethylhexyl) Phthalate, Epigenetic Age Acceleration, and Susceptibility to Early Childhood Upper Respiratory Infections
Source: Epigenomes. 2024 Jan 26;8(1):3. doi: 10.3390/epigenomes8010003 (PMC10885049; doi:10.3390/epigenomes8010003)
Supplement: Supplementary file 1 [file epigenomes-08-00003-s001.zip › epigenomes-2781181-supplementary.pdf]

Sex-Specific Associations between Prenatal Exposure to Di(2-ethylhexyl) Phthalate, Epigenetic Age Acceleration, and Susceptibility to Early Childhood Upper Respiratory Infections

Sarah M. Merrill [1,2,3], Nicole Letourneau [4,5,6,7,8], Gerald F. Giesbrecht [6,7,9,10], Karlie Edwards [2,3], Julia L. MacIsaac [2,3], Jonathan W. Martin [11], Amy M. MacDonald [12], David W. Kinniburgh [12,13], Michael S. Kobor [2,3,14], Deborah Dewey [6,7,8,10], Gillian England-Mason [6,7,*] and The APrON Study Team [15,16,†]

1 Department of Psychiatry and Human Behavior, The Warren Alpert Medical School at Brown University, Providence, RI 02903, USA
2 Department of Medical Genetics, British Columbia Children's Hospital Research Institute, University of British Columbia, Vancouver, BC V6T 1Z4, Canada
3 Centre for Molecular Medicine and Therapeutics, Vancouver, BC V6H 0B3, Canada
4 Faculty of Nursing, University of Calgary, Calgary, AB T2N 1N4, Canada
5 Department of Psychiatry, Cumming School of Medicine, University of Calgary, Calgary, AB T2N 1N4, Canada
6 Department of Pediatrics, Cumming School of Medicine, University of Calgary, Calgary, AB T2N 1N4, Canada
7 Owerko Centre, Alberta Children's Hospital Research Institute, University of Calgary, Calgary, AB T2N 1N4, Canada
8 Hotchkiss Brain Institute, Calgary, AB T2N 4N1, Canada
9 Department of Psychology, Faculty of Arts, University of Calgary, Calgary, AB T2N 1N4, Canada
10 Department of Community Health Sciences, Cumming School of Medicine, University of Calgary, Calgary, AB T2N 1N4, Canada
11 Science for Life Laboratory, Department of Environmental Science, Stockholm University, 106 91 Stockholm, Sweden
12 Alberta Centre for Toxicology, University of Calgary, Calgary, AB T2N 1N4, Canada;
13 Department of Laboratory Medicine and Pathology, University of Alberta, Edmonton, AB T6G 2R3, Canada
14 Program in Child and Brain Development, Canadian Institute for Advanced Research, Toronto, ON M5G 1M1, Canada
15 University of Calgary, Calgary, AB T2N 1N4, Canada
16 University of Alberta, Edmonton, AB T6G 2R3, Canada
* Correspondence: gillian.englandmason@ucalgary.ca
† Membership of the Group is provided in the Acknowledgments.

Figure S1. Flowchart of participants included in the present study.

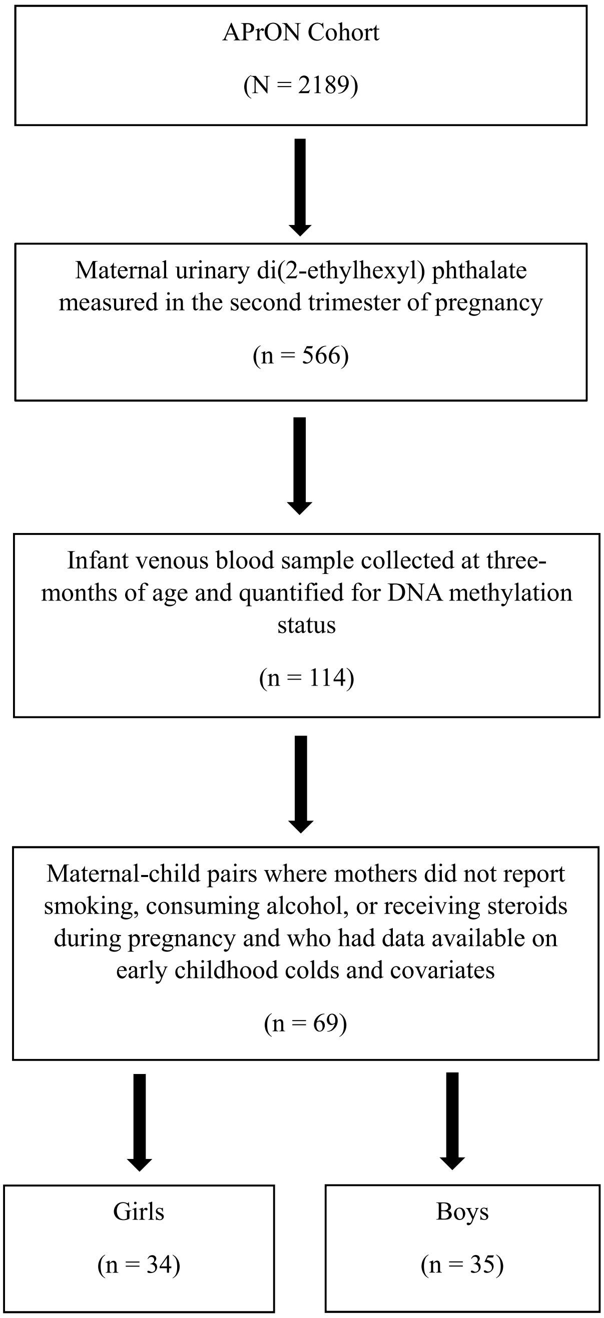

Figure S2 Estimated cell type proportions in three-month-old infant venous blood were not associated with prenatal DEHP exposure across the entire cohort (n=69). Each graph represents the association between each of the 12 estimated cell type proportions (on the y-axis) and DEHP exposure (on the x-axis). On each graph is the Pearson's correlation (*R*) and *p*-value of the correlation between estimated cell type proportion and DEHP exposure.

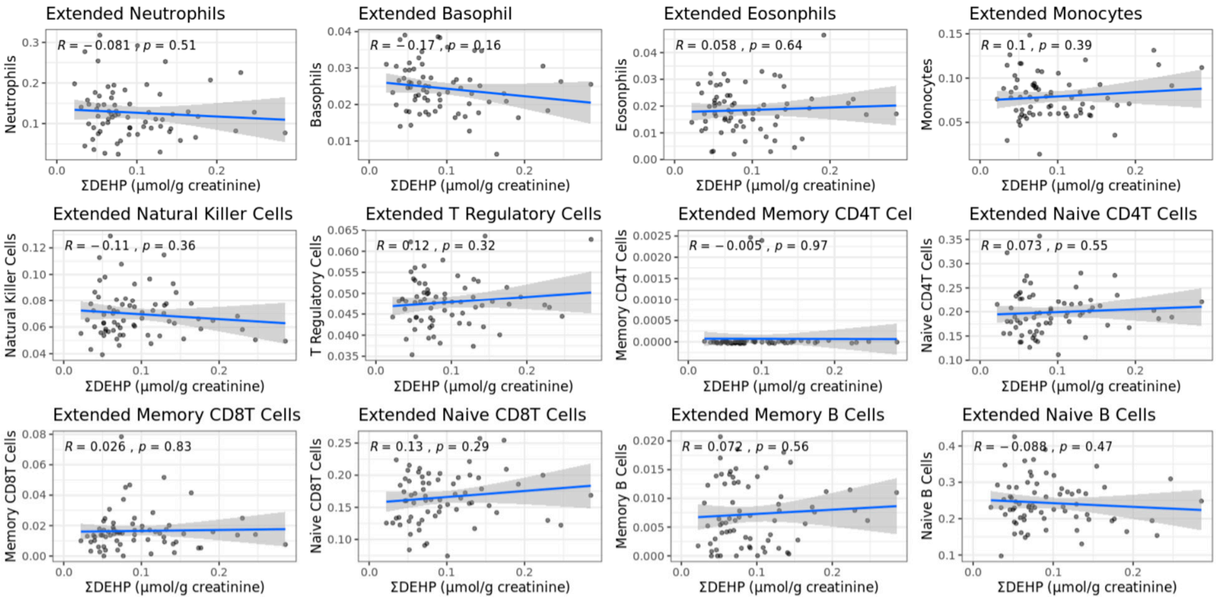

Figure S3 Estimated cell type proportions in three-month-old infant venous blood were not associated with childhood upper respiratory infections (URIs) across the entire cohort (n=69). Each graph represents the association between each of the 12 estimated cell type proportions (on the y-axis) and childhood URIs (on the x-axis). On each graph is the Pearson's correlation (*R*) and *p*-value of the correlation between estimated cell type proportion and childhood URIs.

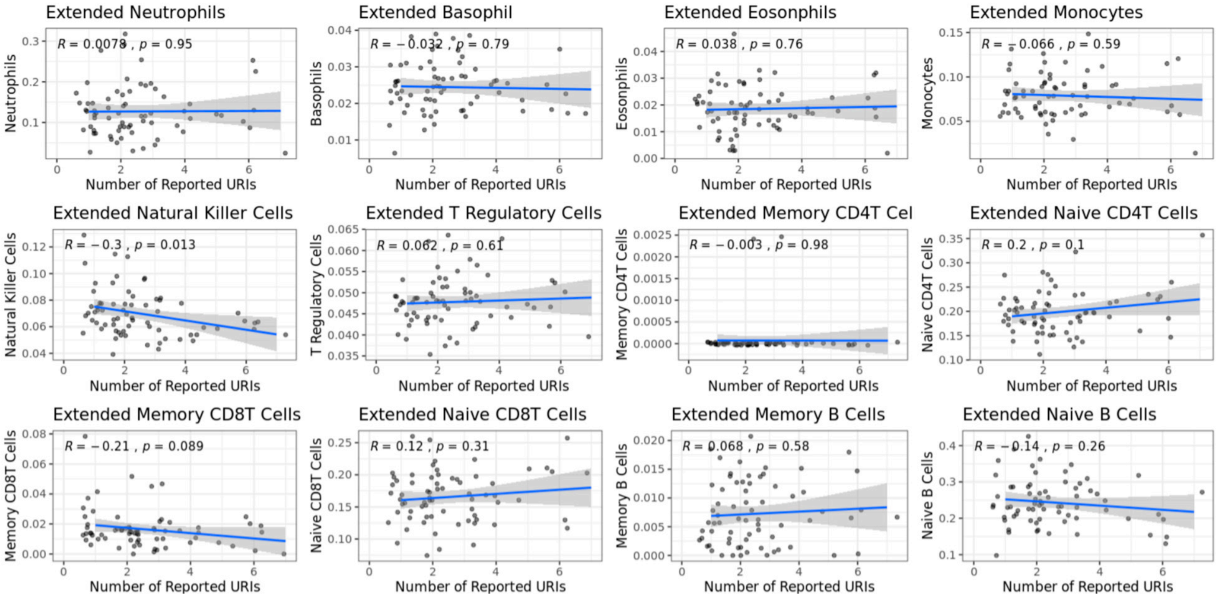

Figure S4 Estimated cell type proportions in three-month-old infant girls' venous blood were not associated with prenatal DEHP exposure (n=34). Each graph represents the association between each of the 12 estimated cell type proportions (on the y-axis) and DEHP exposure (on the x-axis). On each graph is the Pearson's correlation (*R*) and *p*-value of the correlation between estimated cell type proportion and DEHP exposure.

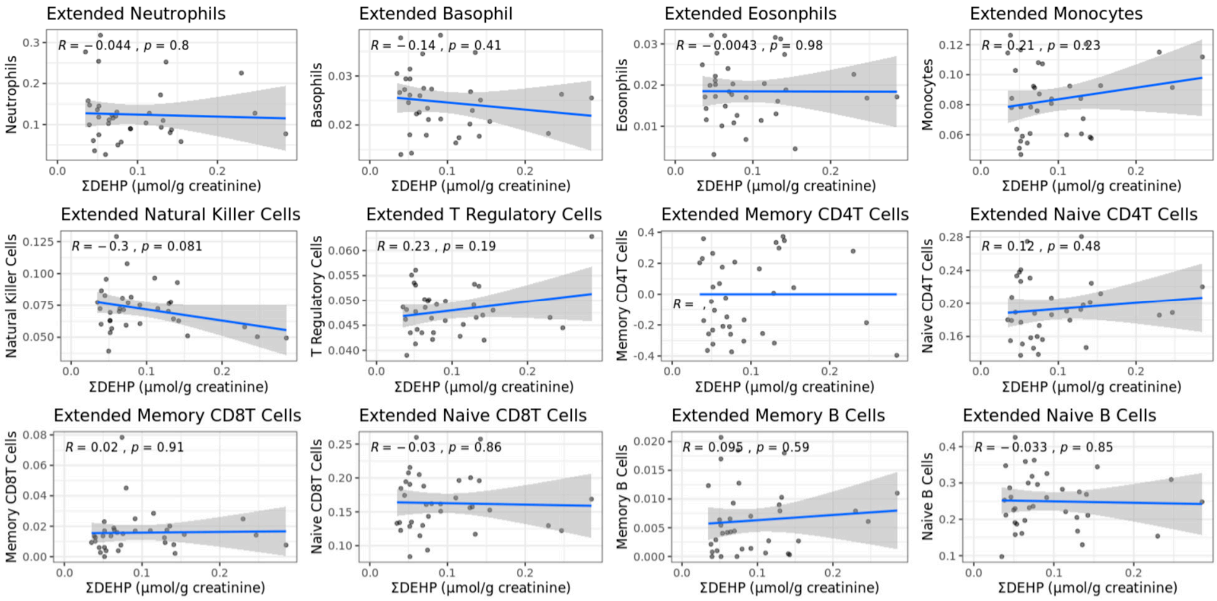

Figure S5 Estimated cell type proportions in three-month-old infant girls' venous blood were not associated with childhood upper respiratory infections (URIs) (n=34). Each graph represents the association between each of the 12 estimated cell type proportions (on the y-axis) and childhood URIs (on the x-axis). On each graph is the Pearson's correlation (*R*) and *p*-value of the correlation between estimated cell type proportion and childhood URIs.

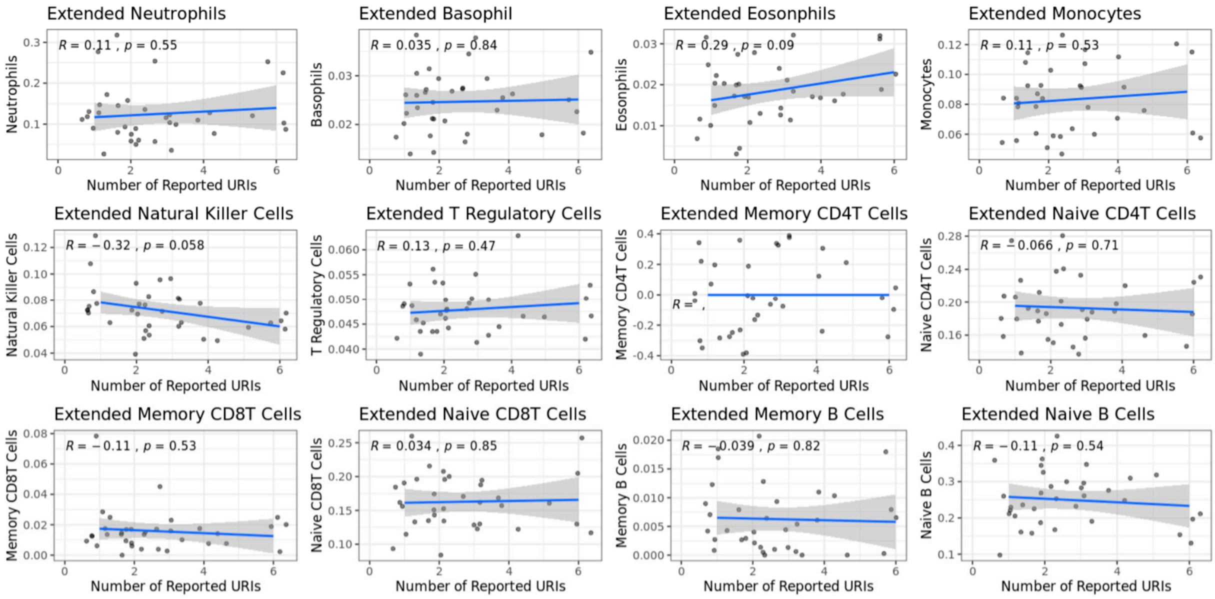

Figure S6 Estimated cell type proportions in three-month-old infant boys' venous blood were not associated with prenatal DEHP exposure (n=35). Each graph represents the association between each of the 12 estimated cell type proportions (on the y-axis) and DEHP exposure (on the x-axis). On each graph is the Pearson's correlation (*R*) and *p*-value of the correlation between estimated cell type proportion and DEHP exposure.

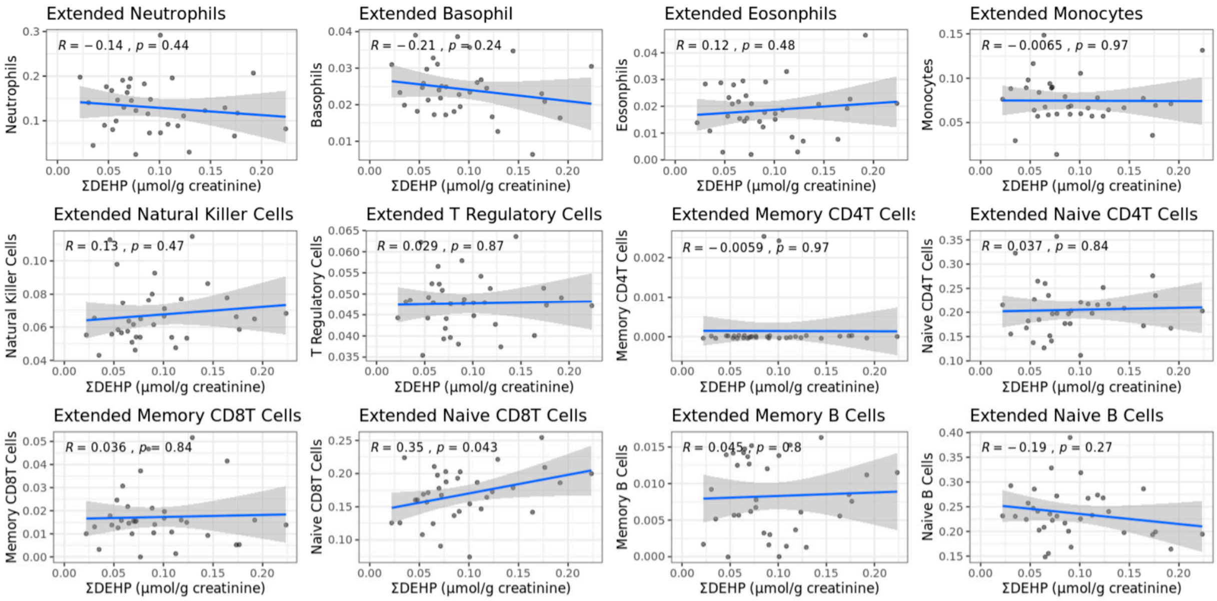

Figure S7 Estimated cell type proportions in three-month-old infant boys' venous blood were not associated with childhood upper respiratory infections (URIs) (n=35). Each graph represents the association between each of the 12 estimated cell type proportions (on the y-axis) and childhood URIs (on the x-axis). On each graph is the Pearson's correlation (*R*) and *p*-value of the correlation between estimated cell type proportion and childhood URIs.

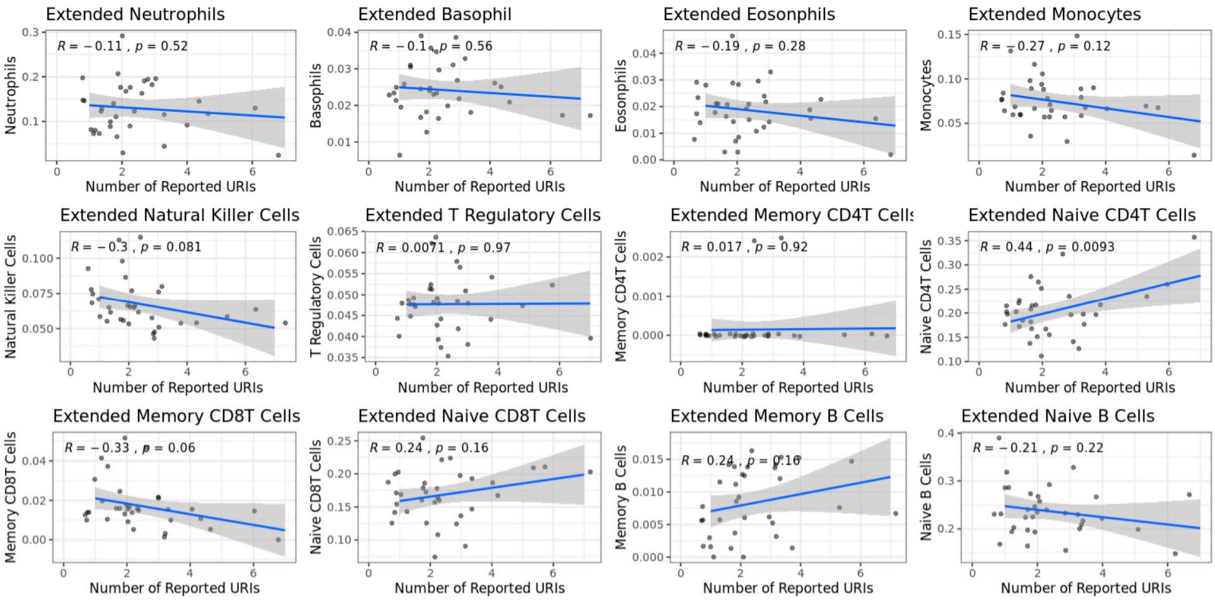

Figure S8. Histogram with kernel density plot of epigenetic age acceleration (EAA) values estimated from the Horvath pan-tissue clock in these samples. The dashed line indicates the mean, which is centered at approximately zero, and the data are approximately normally distributed.

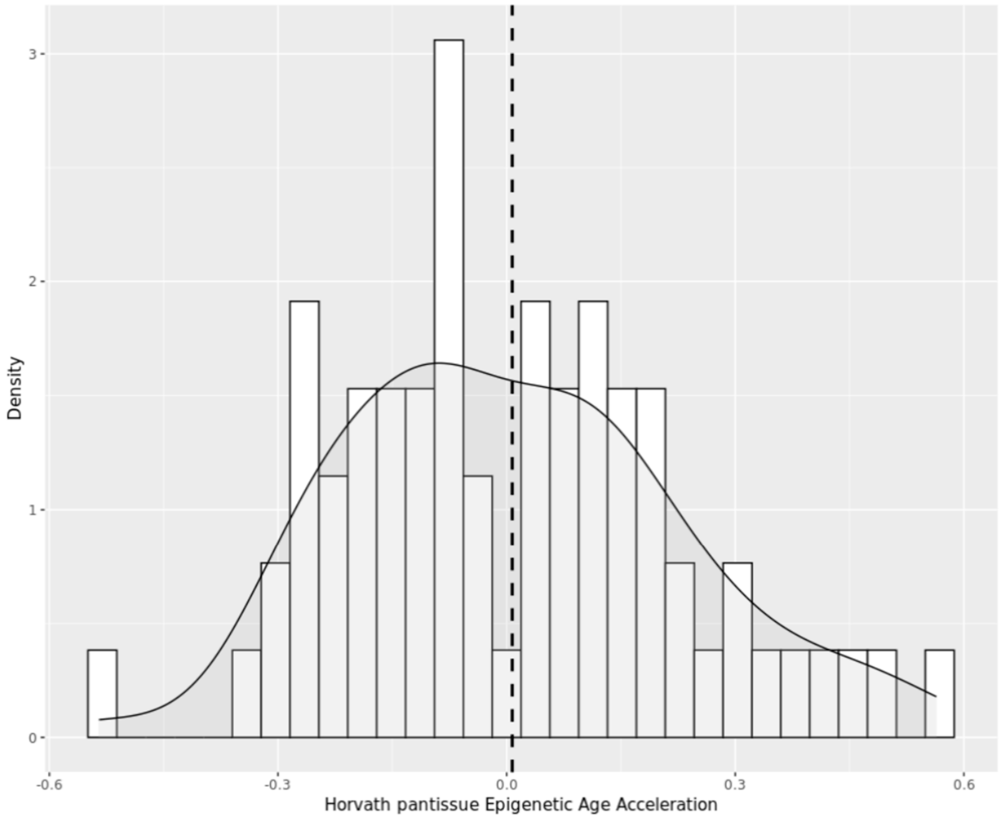

Figure S9. Estimated epigenetic age acceleration (EAA) and epigenetic age difference from the Horvath pan-tissue clock were the same in this sample due to the narrow age range during blood sample collection. The Horvath pan-tissue EAA was derived from residuals of a regression of chronological age on predicted biological age is on the x-axis. While Horvath pan-tissue epigenetic age difference was derived from the difference of chronological age minus predicted biological age. As the correlation is so high, these measures of EAA are synonymous in this sample.

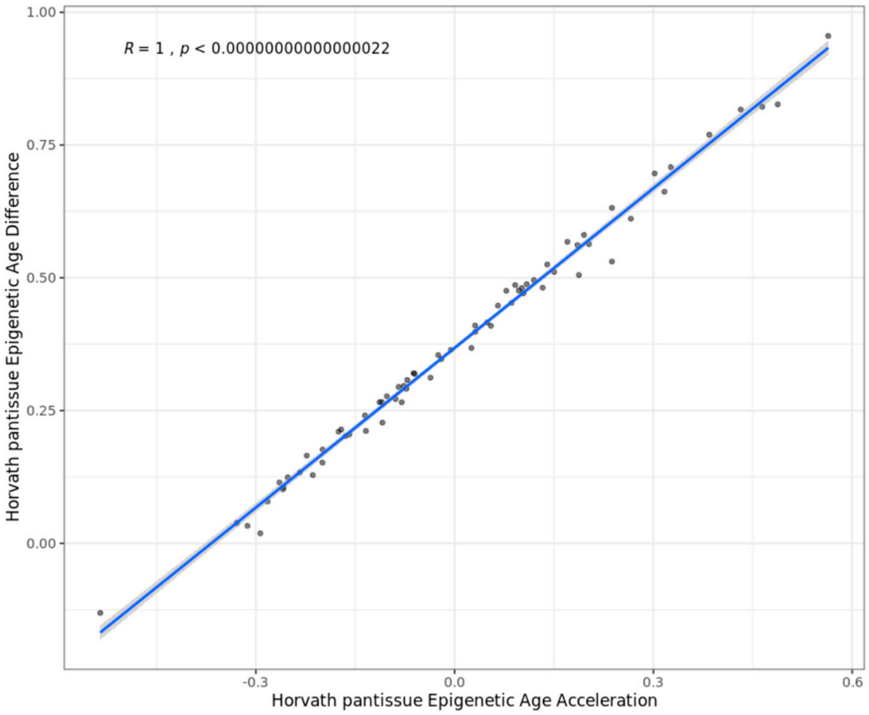

Table S1. Maternal and child characteristics for the sub-sample stratified by child sex (n = 34 girls).

	n (%)	Mean (SD)
**Maternal Characteristics**		
Age (years)	-	30.42 (4.01)
White	31 (91.18%)	-
Married/Cohabiting	34 (100.00%)	-
Household Income > $CAD 70k[1]	27 (79.41%)	-
**Child Characteristics**		
Birthweight (g)	-	3356.29 (389.66)
Gestational age at birth (weeks)	-	39.52 (1.35)
Age at blood draw (weeks)	-	12.52 (1.02)
Number of colds	-	2.42 (1.44)

[1] CAD (Canadian dollars).

Table S2. Maternal and child characteristics for the sub-sample stratified by child sex (n = 35 boys).

	n (%)	Mean (SD)
**Maternal Characteristics**		
Age (years)	-	32.03 (3.81)
White	33 (94.29)	-
Married/Cohabiting	35 (100.00%)	-
Household Income > $CAD 70k[1]	29 (82.86)	-
**Child Characteristics**		
Birthweight (g)	-	3599.03 (578.47)
Gestational age at birth (weeks)	-	39.86 (1.47)
Age at blood draw (weeks)	-	12.52 (0.92)
Number of colds	-	2.66 (1.59)

[1] CAD (Canadian dollars).

Table S3. Creatinine-adjusted prenatal phthalate metabolite concentrations (µg/g creatinine) and DEHP (µmol/g creatinine) in maternal second trimester urine for the sex-stratified groups.

Metabolite	% > LOD	Minimum	Maximum	GM	25th Percentile	50th Percentile	75th Percentile
**Girls (n = 34)**							
MEHP [1]	100%	0.712	13.1	3.34	1.93	3.78	6.27
MEHHP [1]	100%	2.32	33.5	11.0	8.17	10.9	15.0
MEOHP [1]	100%	2.59	22.3	9.27	6.72	9.26	12.8
MECPP [1]	100%	6.41	41.5	16.7	12.0	16.5	18.7
DEHP [2]	-	0.0222	0.224	0.0824	0.0586	0.0810	0.119
**Boys (n = 35)**							
MEHP [1]	100%	1.77	31.0	3.72	2.31	3.03	4.48
MEHHP [1]	100%	3.79	30.7	10.3	6.60	9.42	16.6
MEOHP [1]	100%	4.08	25.7	8.99	5.65	8.28	13.7
MECPP [1]	100%	7.45	62.4	16.6	11.9	15.3	23.4
DEHP [2]	-	0.0350	0.285	0.0805	0.0516	0.0728	0.130

[1] LOD = 0.10 µg/L. [2] molar sum of individual DEHP metabolites.

Note. LOD, limit of detection; GM, geometric mean; MEHP, mono(2-ethyl-hexyl) phthalate; MEHHP, mono(2-ethyl-5-hydroxy-hexyl) phthalate; MEOHP, mono(2-ethyl-5-oxyohexyl) phthalate; MECPP, mono(2-ethyl-5-carboxypentyl) phthalate; ΣDEHPs, sum of low di(2-ethylhexyl) phthalate.

Table S4. Sensitivity analysis adjusting for prenatal exposure to bisphenol A (BPA).

	B **(95% CI)**
**Overall (n = 69)**	
Total Effect (path c)	6.20† (1.01, 11.39)
Path a	-0.23 (-1.23, 0.77)
Path b	3.08† (1.80, 4.36)
Direct effect (path c′)	7.09† (1.86, 12.31)
Indirect effect (path ab)	-1.40 (-5.00, 1.61)
**Girls (n = 34)**	
Total Effect (path c)	-5.79 (-14.56, 2.99)
Path a	-2.32† (-3.54, -1.10)
Path b	1.45 (-1.45, 4.35)
Direct effect (path c′)	-4.03 (-16.09, 8.03)
Indirect effect (path ab)	-3.32 (-8.82, 2.28)
**Boys (n = 35)**	
Total Effect (path c)	9.01† (1.64, 16.39)
Path a	0.90 (-0.56, 2.35)
Path b	3.66† (1.74, 5.78)
Direct effect (path c′)	7.13 (-0.23, 14.49)
Indirect effect (path ab)	3.36 (-0.63, 8.21)

† $p < 0.05$.
